# Glyco-engineering for the production of recombinant IgA1 with distinct mucin-type O-glycans in plants

**DOI:** 10.1080/21655979.2016.1201251

**Published:** 2016-06-22

**Authors:** Martina Dicker, Daniel Maresch, Richard Strasser

**Affiliations:** a Department of Applied Genetics and Cell Biology, University of Natural Resources and Life Sciences, Vienna, Austria; b Department of Chemistry, University of Natural Resources and Life Sciences, Vienna, Austria

**Keywords:** IgA nephropathy, immunoglobulin A, glyco-engineering, glycosylation, O-glycan, post-translational modification, recombinant protein

## Abstract

IgA nephropathy (IgAN) is a common autoimmune disease that is characterized by formation and deposition of IgA1-containing immune complexes frequently leading to end-stage kidney disease. The IgA1 in these immune complexes carries aberrantly glycosylated O-glycans. In circulating IgA1 these galactose-deficient mucin-type O-glycans are bound by autoantibodies and thus, contribute to immune complex formation and pathogenesis. Even though the disease is associated with the overproduction of aberrant O-glycans on IgA1, specific structure-function-studies of mucin-type O-glycans are limited. Compared to other expression hosts, plants offer the opportunity for *de novo* synthesis of O-glycans on recombinant glycoproteins as they are lacking the mammalian O-glycosylation pathway. Recently, we demonstrated that *Nicotiana benthamiana* are suitable for the generation of distinct O-glycans on recombinant IgA1. Here, we expand our engineering repertoire by *in planta* generation of galactose-deficient and α2,6-sialylated O-glycans which are the prevailing glycans detected on IgA1 from patients with IgAN.

## Introduction

Glycosylation is a common post-translational modification that is important for many biological properties including protein folding, stability and protein-protein interactions.^1,2^ The most prominent form of protein glycosylation is the linkage of a glycan to an asparagine (N-glycosylation) on newly synthetized proteins. The other abundant type of glycosylation of secreted and membrane bound proteins is O-glycosylation of serine or threonine residues. Notably, both types of glycosylation are altered in patients with different diseases^2^ and distinct glycans on recombinant glycoprotein therapeutics exhibit optimized efficacy.^3^ N-glycosylation of the Fc domain from human IgG has, for example, a profound influence on effector functions like antibody dependent cell-mediated cytotoxicity.^4^ Whereas, a lot is known about the biological role of N-glycans, less effort has been put in structure-function studies of O-glycans. Indeed, when produced in current mammalian expression systems, glycan-structure-function studies are impeded by the high heterogeneity of the resulting O-glycans.^5^ Obviously, distinct, tailor-made O-glycans on recombinant glycoproteins are required to gain more insight into the function of this type of protein modification. Glyco-engineering of expression hosts has been successfully used to produce recombinant glycoproteins with customized N- and O-glycans.^6,7^ These engineered glycoproteins display enhanced functions and facilitate the development of novel diagnostic tools for detection of aberrant glycosylation. Recently, we showed that *Nicotiana benthamiana* plants are a suitable expression host for the generation of distinct O-glycans on recombinant human IgA1.^8^ In this study, we provide an addition to our O-glycan engineering repertoire and produce a human IgA1 with galactose-deficient and α2,6-sialylated O-glycans. Our engineering approach could be helpful to better understand the influence of distinct O-glycans in IgAN and provide strategies for the development of glycan-related therapeutics.

## Mucin-type O-glycan biosynthesis

**Figure 1.**
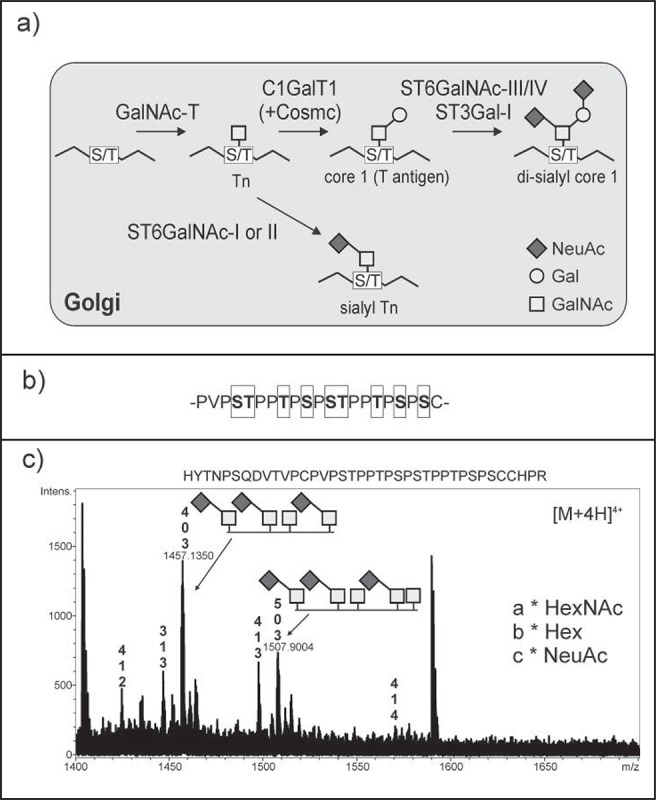
(A) Schematic illustration of the mucin-type O-glycan biosynthesis pathway. Tn and sialyl Tn represent the galactose-deficient O-glycans. GalNAc-T: polypeptide N-acetylgalactosaminyltransferases; C1GalT1: core 1 β1,3-galactosyltransferase; ST6GalNAc-III/IV: α2,6-sialyltransferase III/IV; ST3Gal-I: α2,3-sialyltransferase I; ST6GalNAc-I/II: α2,6-sialyltransferase I/II. Cosmc: core 1 galT1-specific molecular chaperone, required for proper activity of human C1GalT1. (B) IgA1 hinge region. The potential O-glycosylation sites are boxed. (C) *In planta* generation of sialyl Tn structures on the IgA1 hinge region. Human IgA1 was transiently co-expressed with GCSI-GalNAc-T2, GCSI-ST6GalNAc-II and other proteins required for sialylation in *N. benthamiana*. Recombinant IgA1 was purified from leaves, digested with trypsin and analyzed by LC-ESI-MS/MS. The highlighted peaks in the mass spectrum indicate the presence of sialyl Tn and Tn O-glycans on the peptide containing the IgA1 hinge region. Details of protein expression, purification and glycopeptide analysis were described previously.^8^

Depending on the protein-linked sugar there are several types of O-glycosylation. In mucin-type O-glycosylation, N-acetylgalactosamine (GalNAc) is attached to serine or threonine residues.^9^ The initial step of the mucin-type O-glycan biosynthesis in mammals is mediated mainly in the Golgi by polypeptide N-acetylgalactosaminyltransferases (GalNAc-T). The human GalNAc-T family consists of 20 enzymes which display differences in expression in cells and tissues as well as an individual substrate specificity.^9^ The resulting GalNAcα-Ser/Thr is known as the Tn antigen (Fig. 1A). Elongations of the Tn antigen are typically performed by glycosyltransferases in the Golgi of mammalian cells. The common T antigen (also classified as core 1 structure: Galβ1–3GalNAcα-Ser/Thr) is synthesized by core 1 β1,3-galactosyltransferase (C1GalT1). Proper expression of active human C1GalT1 is dependent on the specific molecular chaperon Cosmc which promotes folding of human C1GalT1.^10^ In mammals, core 1 O-glycans are often further modified with sialic acid (N-acetylneuraminic acid: NeuAc) by α2,3- (e.g. ST3Gal-I) and α2,6-sialyltransferases (e.g., ST6GalNAc-III) resulting in the formation of mono- and di-sialylated core 1 O-glycans (Fig. 1A).

## IgA glycosylation and implications for IgA nephropathy

IgAs are the most prevalent antibody class at mucosal sites in the human body and have an important role in the protection of mucosal surfaces from pathogens.^3^ There are 2 subclasses of IgA, IgA1 and IgA2. Human IgA1 exists in 2 versions, the monomeric form, mostly found in circulation and the secreted IgA1 form in which 2 antibody monomers are covalently linked to the joining chain (J-chain).^11,12^ For secretion and transport from the basolateral to the apical pole of exocrine epithelial cells, J-chain-containing IgA1 binds to the polymeric Ig surface-receptor, is released as secretory IgA1 by proteolytic cleavage leaving a glycosylated polypeptide chain, the secretory component, wrapped around the dimeric IgA1.^3,11,12^ Human monomeric IgA1 harbours 2 conserved N-glycosylation sites, one in the CH2 domain and the second one in the C-terminal tailpiece of the α heavy chain. In contrast to IgA2, IgA1 carries an extended hinge region with 9 O-glycosylation sites located between the constant region domains CH1 and CH2 (Fig. 1B). In healthy individuals, 6 of the 9 O-glycosylation sites of IgA1 are typically occupied by mono- and di-sialylated core 1 structures. Truncated, galactose-deficient mucin-type O-glycans without (Tn antigen) or with terminal sialic acid (sialyl Tn: NeuAcα2–6GalNAcα-Ser/Thr) are observed in diseases.^2^ Decreased expression or activity of C1GalT1 or its chaperon Cosmc and enhanced expression or activity of N-acetylgalactosaminide α2,6-sialyltransferase I/II (ST6GalNAc-I/II), the enzymes responsible for sialyl Tn formation, are assumed as possible reasons for the presence of aberrantly glycosylated proteins.^13^

IgAN is seen as the most prevalent form of glomerulonephritis, but the pathogenesis is not well defined.^14^ In IgAN patients, the increased occurrence of galactose-deficient O-glycans is a consistently observed abnormality.^15,16,17^ These truncated Tn or sialyl Tn O-glycans serve as epitopes for antiglycan autoantibodies leading to glomerular immune complex deposits. While the analysis of IgA1 from serum of IgAN patients has made tremendous progress by the use of high-resolution mass spectrometry, the assignment of the influence of the sialyl Tn and Tn antigen formation to the onset of the disease has remained controversial so far. Several studies showed that the galactose-deficient version of IgA1 leads to the formation of immune complexes and therefore has a critical impact on the pathogenesis of IgAN.^18,19^ In contrast, a recent study concluded that the expression of the Tn antigen alone was not sufficient to cause the disease as it was found in patients as well as in healthy individuals and only the level of the total IgA1 concentration in plasma differed significantly between disease and non-disease suggesting that the overexpression of galactose-deficient IgA1 is critical for the disease progression.^16^ Due to these inconsistent data, additional studies are needed to investigate the contribution of the galactose-deficient IgA1 O-glycans and antiglycan antibody immune complex formation to disease pathogenesis.

A possible consequence of aberrant O-glycans might be an influence on the physicochemical properties of the antibody which increases the tendency to aggregate in glomeruli.^20^ The biological roles of sialic acids clustered in O-glycans of IgA1 are of great interest in IgAN but are currently not fully understood.^17^ Serum IgA1 in healthy controls was found to exhibit more α2,3-sialylated O-glycans than α2,6-sialylated glycans which led to the assumption that distribution and the sites of sialic acid attachment are critically involved in the pathogenesis of IgAN. Apart from that, hypersialylation of IgA1 influences the overall negative charge of the protein leading to enhanced affinity of the antibody to mesangial cells.^21^ A reduced level of sialic acid and galactose in the IgA1 hinge region was suggested to increase the affinity to extracellular matrix proteins.^22^ Moreover, sialylated glycans cannot bind to the hepatic asialoglycoprotein receptor which reduces the rate of clearance and prolongs the persistency of immune complexes in the circulation.^23^ In summary, the ill-defined and even controversial impact of distinct IgA1 O-glycans in IgAN requires further characterization of the carbohydrate structures in order to understand the mechanisms underlying the pathogenesis of IgAN and the production of recombinant IgA1 with defined mucin-type O-glycans that can be used in disease models.^17^

## Engineering of mucin-type-O-glycans on recombinant IgA1 in *N. benthamiana* plants

In order to get more insight into the pathogenesis of diseases like IgAN, tailored and homogenous mucin-type O-glycans are required to perform specific structure-function studies.^14^ For that purpose, production hosts other than mammalian cells appear highly suitable. Plants do not show a typical mucin-type O-glycosylation pathway which provides the opportunity for *de novo* synthesis. However, plants can convert proline residues adjacent to Ser/Thr O-glycosylation sites into hydroxyproline.^8^ The hydroxyproline residues can then be further extended with arabinose chains or huge arabinogalactans and neighboring serine residues can be modified with a single galactose residue.^24,25^ Such plant-specific modifications are unwanted and can be eliminated by knockout of the responsible enzymes like prolyl 4-hydroxylases. This engineering approach has been successfully carried out in a moss-based expression system^26^ and similar strategies are currently developed for higher plants like *N. benthamiana*.

To produce a recombinant human IgA1 with defined mucin-type O-glycans in *N. benthamiana* we transiently expressed different mammalian glycosyltransferases and proteins required for the biosynthesis and transport of the precursor CMP-sialic acid together with the recombinant glycoprotein.^8^ Mucin-type O-glycan formation was efficiently initiated by co-expression of human GalNAc-T2. Since expression of human C1GalT1 with its chaperone Cosmc did not result in any detectable core 1 O-glycan formation,^27^ we expressed *Drosophila melanogaster* C1GalT1 which is functional in the absence of a Cosmc-like chaperone. Further expression of human α2,3-sialyltransferase and *Mus musculus* α2,6-sialyltransferase resulted in the formation of sialylated core 1 O-glycans that are normally found in the hinge region of human IgA1.^8^ Our study does not only demonstrate the potential of *N. benthamiana* for the production of tailored mucin-type O-glycans on therapeutically interesting proteins but raise also the opportunity for the generation of pathologically relevant modifications, like the sialyl Tn antigen found on circulating IgA1 in IgAN patients. To this end, we examined whether the co-expression of the sialic acid pathway with human GalNAc-T2 and ST6GalNAc-II will result in the *in planta* generation of sialyl Tn on recombinant IgA1. The binary vectors required for the expression of recombinant human IgA1, the proteins for the initiation of mucin-type O-glycosylation, CMP-sialic acid biosynthesis as well as transport were described in detail previously.^27,28,8^ As we wanted to avoid the aforementioned plant-specific O-glycosylation which mainly takes place in the Golgi we used ER-retained versions of GalNAc-T2 and ST6GalNAc-II. Retention of glycosyltransferases and glycosidases in the ER by exchanging the N-terminal targeting regions by that of the ER-resident transmembrane protein α-glucosidase I (GCSI) has been performed previously by our group for GalNAc-T2.^29^ The GCSI-ST6GalNAc-II construct was generated in a similar way. Following co-expression of IgA1 with ER-retained GCSI-GalNAc-T2, GCSI-ST6GalNAc-II and with the machinery for CMP-sialic acid production and transport, IgA1 was purified, subjected to SDS-PAGE and the corresponding heavy chain band was digested with trypsin. LC-ESI-MS/MS analysis revealed peaks corresponding to glycopeptides with 3 O-glycosylation sites occupied by the sialyl Tn structure and one or 2 additional sites occupied with the Tn antigen (Fig. 1C). Apart from that minor peaks were present with an additional single hexose indicating the presence of small amounts of plant-specific O-glycosylation.

## Conclusion and prospects

In addition to our recently plant-produced core 1 structures on recombinant IgA1,^8^ we have provided now an approach for tailored sialylation of GalNAc-Ser/Thr O-glycans on human IgA1, also identifying human ST6GalNAc-II as a suitable sialyltransferase for this *in vivo* engineering strategy. Further optimization of targeted O-glycan modifications can be achieved by the use of multi-gene expression cassettes, stable transformed plants and fine-tuned subcellular targeting of non-plant glycosyltransferases. Implementation of these tools will result in the generation of an expression platform that is optimally suited for the reconstruction of physiologic or non-physiologic O-glycan structures on recombinant proteins. This may facilitate a better understanding of disease progression and can lead to the development of glycan-related biomarkers to monitor disease stages.^30^ Notably, no specific IgAN therapy is currently available. Small glycoproteins or glycopeptides mimicking the galactose-deficient IgA1 hinge region can be produced in our glyco-engineered plants. Such glycopeptides could be tested in a disease-specific therapy to block the formation of IgA1-antiglycan immune complexes.
